# Effect of the combination of 25-hydroxyvitamin D3 and higher level of calcium and phosphorus in the diets on bone 3D structural development in pullets

**DOI:** 10.3389/fphys.2023.1056481

**Published:** 2023-04-24

**Authors:** Dima White, Chongxiao Chen, Woo Kyun Kim

**Affiliations:** Department of Poultry Science, University of Georgia, Athens, GA, United States

**Keywords:** 25-hydroxyvitamin D 3, bone 3D structure, pullets, micro-CT (μCT) scanning technology, bone health

## Abstract

Bone issues such as osteoporosis are major concerns for the laying hen industry. A study was conducted to improve bone-health in pullets. A total of 448 one-day-old Hyline W36 pullets were randomly assigned to four treatments (8 rep; 14 birds/rep) until 17 weeks (wks). Dietary treatments were: 1) vitamin D_3_ at (2,760 IU/kg) (D), 2) vitamin D_3_ (2,760 IU/kg)+62.5 mg 25-(OH)D_3_/ton (H25D), 3) vitamin D_3_ (2,760 IU/kg) + 62.5 mg 25-(OH)D_3_/ton + high Ca&P (H25D + Ca/P), and 4) vitamin D_3_ (2,760 IU/kg) + high Ca&P (D + Ca/P). The high calcium (Ca) and phosphorus (P) diet was modified by increasing both high calcium and phosphorus by 30% (2:1) for the first 12 wks and then only increasing P for 12–17 wks to reduce the Ca to P ratio. At 17 wk, growth performance was measured, whole body composition was measured by dual energy x-ray absorptiometry (DEXA), and femur bones were scanned using Micro-computed tomography (Micro-CT) for bone 3D structure analyses. The data were subjected to a one-way ANOVA using the GLM procedure, with means deemed significant at *p* < 0.05. There was no significant outcome for growth performance or dual energy x-ray absorptiometry parameters. Micro-computed tomography results indicated that the H25D + Ca/P treatment had lower open pore volume space, open porosity, total volume of pore space, and total porosity in the cortical bone compared to the D + Ca/P. It also showed that a higher cortical bone volume/tissue volume (BV/TV) in the H25D + Ca/P than in the D + Ca/P. Furthermore, the H25D + Ca/P treatment had the lowest trabecular pattern factor and structure model index compared to the other treatments, which indicates its beneficial effects on trabecular structural development. Moreover, the H25D + Ca/P had a higher trabecular percentage compared to the D and 25D, which suggests the additional high calcium and phosphorus supplementation on top of 25D increased trabecular content in the cavity. In conclusion, the combination of 25D with higher levels of high calcium and phosphorus could improve cortical bone quality in pullets and showed a beneficial effect on trabecular bone 3D structural development. Thus, combination of a higher bio-active form of vitamin D_3_ and higher levels of high calcium and phosphorus could become a potential feeding strategy to improve bone structural integrity and health in pullets.

## Introduction

The ever-increasing improvement in laying hen nutrition and genetics has resulted in vastly more efficient hens capable of laying more than 340 eggs by 72 weeks of age (Lohmann, 2016; Preisinger, 2018). With advancements challenges, come with increased susceptibility to bone problems as demonstrated by osteoporosis and bone fractures in laying hens (Lohmann, 2016). Therefore, a better understanding of bone modeling; restoration before or during egg laying periods would be crucial to address bone development and health issues. One of the physiological changes in laying hens is that the structural bone development ceases at sexual maturity, meaning that the elongation, widening, and mineralization of structural bones (cortical and trabecular) discontinue when laying starts (Whitehead, 2004; Kim et al., 2012; Chen et al., 2020a). Hens lay eggs continuously without molting, suggesting no opportunity for the possibility of cortical bone regeneration once laying starts (Whitehead, 2004). Hence, rearing is a vital period for establishing optimal bone mineralization and structural development with implications on eggshell quality and bone health during laying (Regmi et al., 2015; Chen et al., 2020a; Chen et al., 2020b).

Osteoporosis is described as a progressive decrease in the amount of mineralized structural bone and leads to bone fragility and susceptibility to fracture (Kim et al., 2012; Toscano, 2018). These anomalies can also diminish skeletal calcium reserves and contribute to the seriousness of osteoporosis. However, even in the lack of caged layer paralysis, osteoporosis is prevalent in laying flocks (Whitehead and Fleming, 2000) and is a foremost contributory factor in the high incidence (approx. 30%) of hens experiencing fractures (Gregory and Wilkins, 1989; Clark et al., 2008; Kim et al., 2012). Nutritional deficiencies of calcium, phosphorus, or cholecalciferol have been shown to result in bone loss attributable to osteomalacia (Wilson and Duff, 1991) and are likely to lead to greater severity of osteoporosis (Clark et al., 2008; Kim et al., 2012).

Numerous pullet studies have addressed the importance of early bone development, and its prolonged effects on bone health during laying periods (Casey-Trott et al., 2017; Chen et al., 2020a; Chen et al., 2020b; Hester et al., 2013; Kim et al., 2012; Regmi et al., 2015). However, prior nutritional studies seldom focused on the pullet period because osteoporosis was observed during the laying period; it was not successful to reverse it by using nutritional approaches targeting the laying period (Rennie et al., 1997; Kim et al., 2012; Chen et al., 2020a). Thus, exploring early intervention for resolving late issues of osteoporosis in laying hens is recommended.

25-Hydroxyvitamin D_3_ (H25D) is an intermediate form of vitamin D_3_ and is now readily available for commercial use in the poultry industry since 2006 (Adhikari et al., 2020). H25D has been numerously studied in broiler research for enhancing bone health and Ca and P absorption (Kim et al., 2011; Wideman et al., 2015; Han et al., 2016; Świątkiewicz et al., 2017; Chen et al., 2020a; Chen et al., 2020b). However, only few studies on laying hens and especially pullets for developing the healthy bone structures for performance and maintenance during the laying period (Käppeli et al., 2011; Nascimento et al., 2014; Silva, 2017; Adhikari et al., 2020; Chen et al., 2020a; Chen et al., 2020b). The exploration of nutrients simply during a laying phase is too late for finding ways to fix or improve concerns with bone issues that should have been prevented in the rearing period to avoid the ultimate outcome of osteoporosis. Even human clinical trials demonstrated that there was little to no effect of supplementation of vitamin D or calcium on fracture occurrences in elder populations (Zhao et al., 2017).

Previous research has found that an additional 25-(OH)D_3_ in the pullet diet stimulated bone growth, increased bone size, and created more pores in the cortical bone during a pullet period, which allowed more mineral deposition in the bones during a laying period (Chen et al., 2020a), maintaining better bone structural integrity and health in the later laying period. Due to the larger structure size, the bone mineral density (BMD) was decreased in 25-(OH)D_3_ treatment during the pullet period, and this was possibly attributed to the calcium (Ca) and available phosphorous (P) level and ratio in the diets which were not ideal to provide sufficient Ca and P to induce proper mineral deposition in increased bone size by H25D (Chen et al., 2020a). Thus, increasing minerals, such as calcium and phosphorus in addition to H25D supplementation in rearing diets, can ultimately develop structurally sound bones and in the long run can possibly improve laying hen performances while maintaining bone health. Therefore, a study was conducted to explore the effect of dietary 25-Hydroxyvitamin D_3_combined with modified Ca and P level and ratio on bone development and mineralization in pullets. The objective was to evaluate the effect of H25D combined with modified Ca and P level and ratio in the diets on growth performance, body composition, and bone 3D structural development in pullets.

## Materials and methods

### Animal and housing

The study was conducted at the research facility of the Department of Poultry Science at the University of Georgia. The trial was conducted in accordance with the Institutional Animal Care and Use Committee at the University of Georgia. A total of 448 one-day-old Hyline W36 chicks were randomly assigned to 4 dietary treatments (8 replicates and14 birds/replicate) and raised until 17 wks. Chicks were obtained from at Hy-line North America hatchery (Mansfield, GA). Birds were housed in colony cages for 17 wks [(90 cm (L) × 46 cm (W) × 38 cm (H)]. Water and experimental diets were offered *ad libitum* from 0 to 17 wks. There were two nipples per cage. The pullets received an intermittent lighting program during the first 7 days with 4 h of light followed by 2 h of dark circles. The lighting management was customized by Hy-line North America lighting program throughout 2–17 wks (http://sales.hyline.com/NALighting/WebLighting.aspx).

### Experimental diets

The diets were designed based on the Hy-Line W36 guide (2020). The formulation of corn-soybean based diet is shown in Table 1. Treatments consisted of: 1) control diet; vitamin D_3_ at (2,760 IU/kg)IU/kg (D), 2) vitamin D_3_ (2,760 IU/kg) + 62.5 mg 25-(OH)D_3_/ton (H25D), 3) vitamin D_3_ (2,760 IU/kg) + 62.5 mg 25-(OH)D_3_/ton + high Ca and P (H25D + Ca/P), and 4) vitamin D_3_ (2,760 IU/kg) + high Ca and P (D + Ca/P). The high Ca and P diet was modified by increasing 30% of both Ca and P (2:1) for the first 12 wks, and then only increasing P for 12–17 wks to reduce the Ca to P ratio (Table 2). The experimental diets were fed for 17 wks. The diets were freshly mixed every 3 weeks to minimize degradation of supplemented vitamin D_3_ or 25-(OH)D_3_ in the diets.

**TABLE 1:** Ingredients and diet composition for treatments D and H25D of the control diets fed to pullets at different stages for 17 weeks.

Ingredients	Starter 1	Starter 2	Grower	Developer	Prelay
Unit %	1–3 weeks	4–6 weeks	7–12 weeks	13–15 weeks	16–17 weeks
Corn	66.63	64.62	70.00	69.43	66.00
Soybean Meal −48%	28.40	25.00	21.62	20.00	21.50
Soybean Oil	1.00	2.66	1.38	2.57	2.34
Limestone	0.68	0.71	0.81	1.95	4.69
Defluor. Phos	2.03	2.03	1.93	1.85	2.01
Common Salt	0.30	0.30	0.30	0.30	0.30
L-Lysine HCl	0.18	0.22	0.71	0.08	0.12
DL-Methionine	0.20	0.25	0.20	0.14	0.21
Threonine	0.10	0.12	0.09	0.05	0.05
Vitamin Premix (T)	0.05	0.05	0.05	0.05	0.05
Mineral Premix	0.06	0.06	0.06	0.06	0.06
Coccidiostat (Ampro)	0.05	0.05	0.05	0.05	0.05
Sand	0.31	3.93	2.79	3.47	2.62
ME (kcal/kg)	3,030	3,030	3,030	3,050	2,960
CP (%)	20.00	18.25	17.50	16.00	16.50
Ca (%)	1.00	1.00	1.00	1.40	2.50
Avail Phos (%)	0.50	0.49	0.47	0.45	0.48
Met (%)	0.51	0.53	0.47	0.40	0.47
Met + Cys (%)	0.83	0.83	0.75	0.67	0.74
Lys (%)	1.15	1.07	1.35	0.83	0.89
Thr (%)	0.82	0.77	0.70	0.62	0.64

^a^ D = vitamin D3 (2,760 IU/kg); H25D = vitamin D3 (2,760 IU/kg) + 62.5 mg HyD/ton (full dose).

^b^ Control diet contains 3,000 IU, of vitamin D3/kg of feed.

^c^ Vitamin premix supplied per kilogram of complete feed: vitamin A, 8,250 IU; vitamin E, 30 IU; vitamin B_12_, 0.013 mg; vitamin K_3_, 2.0 mg; niacin, 23.6 mg; choline chloride, 1,081 mg; folic acid, 4.0 mg; biotin, 0.25 mg; pyridoxine, 4.0 mg; thiamine, 4.0 mg; vitamin D_3,_ 3,000 IU.

^d^ Mineral premix supplied per kg of complete feed: manganese oxide, 70 mg; zinc oxide 80 mg, ferrous sulfate, 80 mg, copper sulfate, 10 mg; sodium selenium, 0.3 mg; calcium iodate premix, 0.5 mg.

^e^ Starter 1 = fed from 1 to 3 wks, starter 2 = fed from 4 to 6 wks, grower = fed from 7 to 12 wks, developer = fed from 13 to 15 wks, prelay = fed from 16–17 wks.

**TABLE 2:** Ingredients and diet composition for treatments H25D + Ca/P and D + Ca/P diets with higher levels of Ca&P fed to pullets at different stages for 17 weeks.

Ingredients	Starter 1	Starter 2	Grower	Developer	Prelay
Unit %	0–3 weeks	3–6 weeks	6–12 weeks	12–15 weeks	15–17 weeks
Corn	65.38	64.62	70.00	69.43	66.00
Soybean Meal −48%	28.64	25.00	21.62	20.00	21.50
Soybean Oil	1.41	2.66	1.38	2.57	2.34
Limestone	0.77	0.80	0.94	0.78	2.59
Defluor. Phos	2.87	2.86	2.71	3.24	4.51
Common Salt	0.30	0.30	0.30	0.30	0.30
L-Lysine HCl	0.18	0.22	0.71	0.08	0.12
DL-Methionine	0.20	0.25	0.20	0.14	0.21
Threonine	0.10	0.12	0.09	0.05	0.05
Vitamin Premix (T)	0.05	0.05	0.05	0.05	0.05
Mineral Premix	0.06	0.06	0.06	0.06	0.06
Coccidiostat (Ampro)	0.05	0.05	0.05	0.05	0.05
Sand	0.00	3.01	1.88	3.25	2.22
Calculated value					
ME (kcal/kg)	3,030	3,030	3,030	3,050	2,960
CP (%)	20.00	18.25	17.50	16.00	16.50
Ca (%) (+30%)	1.30	1.30	1.30	1.40	2.50
Avail Phos (%) (+30%)	0.65	0.64	0.61	0.70	0.93
Met (%)	0.51	0.53	0.47	0.40	0.47
Met + Cys (%)	0.83	0.83	0.75	0.67	0.74
Lys (%)	1.15	1.07	1.35	0.83	0.89
Thr (%)	0.82	0.77	0.70	0.62	0.64
Analyzed value					
HPLC/D (mcg/g)	139.4	146.2	134.3 mcg/g	134.3	139.4
HPLC/H25D (mcg/g)	<6.3	77.3	159.7 mcg/g	159.7	<6.3
Analysis Calcium (%)	1.33	1.34	1.35	1.42	2.55
Analysis Total Phos (%)	0.86	0.84	0.90	1.00	1.17
Analysis Avail Phos (%)	0.66	0.64	0.70	0.80	0.97

^a^ H25D + Ca/P = vitamin D3 (2,760 IU/kg) + 62.5 mg HyD/ton (full dose) + 10%–30% higher Ca&P levels; D + Ca/P = vitamin D3 (2,760 IU/kg) + 10%–30% higher Ca&P levels.

^b^ Control diet contains 3,000 IU, of vitamin D3/kg of feed.

^c^ Vitamin premix supplied per kilogram of complete feed: vitamin A, 8,250 IU; vitamin E, 30 IU; vitamin B_12_, 0.013 mg; vitamin K_3_, 2.0 mg; niacin, 23.6 mg; choline chloride, 1,081 mg; folic acid, 4.0 mg; biotin, 0.25 mg; pyridoxine, 4.0 mg; thiamine, 4.0 mg; vitamin D_3,_ 3,000 IU.

^d^ Mineral premix supplied per kg of complete feed: manganese oxide, 70 mg; zinc oxide 80 mg, ferrous sulfate, 80 mg, copper sulfate, 10 mg; sodium selenium, 0.3 mg; calcium iodate premix, 0.5 mg.

^e^ HPLC, High-performance liquid chromatography.

^f^ Starter 1 = fed from 1 to 3 wks, starter 2 = fed from 4 to 6 wks, grower = fed from 7 to 12 wks, developer = fed from 13 to 15 wks, prelay = fed from 16–17 wks.

### Serum 25-(OH)D_3_ content analysis

Blood samples (8 birds/treatment) were collected from the wing vein at 17 wks. After the blood was clotted, the blood samples were centrifuged at 1,500 g in a refrigerated centrifuge (Eppendorf Centrifuge 5430R; Eppendorf, Hamburg, Germany) for 12 min. The serum was collected and transferred into a clean polypropylene tube. The samples were maintained at −80°C until analysis. The serum 25-(OH)D_3_ level was determined using a mass spectrometry procedure (Heartland Assays, Ames, IA).

### Growth performance measurement

Body weight (BW), body weight gain (BWG), and feed intake (FI) were recorded at 0, 3, 6, 12, 15, and 17 wks. The phase periods for recording are 0–3 wks (Starter 1 period), 4–6 wks (Starter 2 period), 7–12 wks (Grower period), 13–15 wks (Developer period), and 16–17 wks (Peak period). However, growth performance was reported for 0–17 wks.

### Bone quality measurements

At 17 wks, two birds per cage (2 birds × 8 replicates = 16 birds per treatment) were euthanized by cervical dislocation. Dual energy x-ray absorptiometry (DEXA; pDEXA^®^, Bone Densitometer, General Electric Company, 41 Farnsworth Street, Boston, MA 02210, United States) was used for whole bird body composition analyses, and the whole bird was defined as a region of interest (Kim et al., 2004; Chen et al., 2020a; Adhikari et al., 2020). Whole body region scans were conducted to measure bone mineral density (g/cm^2^) (BMD), bone mineral content (g) (BMC), bone area (cm^2^), fat weight (kg), fat percent (%), muscle weight (kg), muscle percentage (%), and total body weight (kg). Each sample bird was placed chest up on the scanner at the same position and orientation during the measurement. All scans were obtained at a scan speed of 2.5 mm/s, with a voxel resolution of 0.07 mm × 0.07 mm × 0.50 mm.

The left femur bone was taken from one bird per pen (8 bones/treatment) at 17 wks of age. After the soft tissue was removed completely, the samples were wrapped with PBS-soaked cheesecloth to keep the bones moist. The femur bones were scanned with micro-computed tomography (Micro-CT; Skyscan 1275; Bruker micro-CT, Kartuizersweg 3B Kontich, Belgium) which was used for 3- dimensional image acquisition (Chen and Kim, 2020; Yamada et al., 2021). The femur bone was held in a low-density 50 ml tube; extra cheesecloth was used for holding the sample in a vertical orientation and firmly inside the container. The container was then mounted on the scanning stage. Scan settings and definitions/explanations of measurements are shown in Table 3. Before the scanning, the alignment test and flat field correction were performed for calibration according to the Bruker Micro-CT manual (Bruker micro-CT, Kartuizersweg 3B Kontich, Belgium). Random movement and 180-degree scanning were applied along with a 0.5 mm aluminum filter used to reduce beam hardening. The x-ray source was set at 80 kV and 125 µA. The pixel size was fixed at 25 μm, the rotation angle of 0.40 was applied at each step, and four images per rotation were captured. A series of 2-D images were capturedand later used to reconstruct a 3-D image using N-Recon (Bruker Micro-CT, Billerica, MA). After scanning, the pictures were carefully screened, and the appropriate alignment and mathematical method correction were used (beam-hardening correction: 35%; smoothing: NA; ring artifact reduction: NA). The 3-D model was then repositioned using Data Viewer software (Bruker Micro-CT, Billerica, MA) to ensure the consistency of selecting the region of interests for the following analysis. The volume of interest was selected using CTAn software (Bruker Micro-CT, Billerica, MA). The volume of interest was defined as the section of the bone from which morphometry and density measurements were analyzed. The bone section chosen was a distal supracondylar region from which a total of 300 slides/frames were analyzed. This 300 slide/frame section is the epiphyseal plate region of the long bone. Two phantoms (8 mm diameter) of known density (0.25 and 0.75 g/cm3) for calcium hydroxyapatite [(Ca5(PO4)3(OH)] were scanned to allow for calibration of bone mineral density (Sharma et al., 2021). The different parts of bones were separated according to the methods described by Chen and Kim (2020).

**TABLE 3:** Micro-CT scanning settings and definitions/explanations.

Voltage	Current	Exposure time	Rotation	Average frame	Pixel size
(kv)	(mA)	(ms)	(degrees)	(Slides)	(Um/pixel)
80	125	55	0.4	6	25
Parameters	Definition/explanation				
Tissue volume	The volume of the volume-of-interest (VOI)				
Bone volume	The volume of binarized objects (mineralized bones) with in the VOI				
Pore number	The total number of closed pores within VOI				
Pore volume	The total volume of pores				
Closed pore	Closed pores in a 3D; is a connected assembly of space, surrounded on all slides in 3D by solid voxels. The measurement includes volume, surface and number				
Opened pore	Defined as any space located within a solid object or between solid objects, which has any connections in 3D to the space outside the object or objects. The measurement includes volume, surface, and number				
Porosity	The volume of pores as a percentage of total VOI volume				
Trabecular connectivity	A 3D calculation based on 2D scale. In simple, higher number means better connected trabecular structure				
Trabecular connectivity density	Connectivity/VOI				
Trabecular pattern Factor	This is an inverse index of connectivity. To simplify, lower number means better connectivity and trabecular structure				
Trabecular thickness	The diameter of the largest sphere which fulfils two conditions: the sphere encloses the point, and the sphere is entirely bound within the solid surfaces. This is mean value of the trabecular thickness in 3D space				
Structure model index	Widely used to measure rods and plates in trabecular bone. It exploits the change in surface curvature that occurs as a structure varies from spherical, to cylindrical to planar. To simplify, lower number indicates better connectivity and trabecular structure				
Degree of anisotropy	Isotropy is a measure of 3D symmetry or the presence or absence of preferential alignment of structures along a particular directional axis. Consider a region or volume containing two phases (solid and space), both having complex architecture, such as a region of trabecular bone. We can study this volume to determine isotropy. Values for DA calculated in this way vary from 1 (fully isotropic) to infinity (fully anisotropic). Higher number indicates positive connectivity which will also correspond with lower TPF and SMI				

### Statistical analysis

Data were subjected to statistical analyses using the general linear model procedure (GLM) of SAS (SAS Institute Inc., Cary, NC). One-way ANOVA was used with model _Yij_ = µ +α_i_ +ε_ij_. Significant differences among the treatments were determined using GLM procedures. Data were considered significantly different at *p* ≤ 0.05, and trends (0.05 ≤ *p* ≤ 0.1) were also presented (Choi et al., 2021).

## Results

In the current study, the supplementation of different isoforms of vitamin D (D or H25D) along with higher Ca and P levels did not have an impact on growth performance parameters (*p* > 0.05) (Table 4) or whole bird body composition parameters (Table 5).

**TABLE 4:** Growth performance at 17th week.

Treatment	BW/Bird (kg)	BWG/Bird (kg)	FI/Bird (kg)
D	1.239	1.202	5.268
H25D	1.229	1.192	5.298
H25D + Ca/P	1.246	1.209	5.266
D + Ca/P	1.248	1.211	5.215
SEM	0.025	0.024	0.027
*p*-value	0.998	0.992	0.302

^a^ BW, Body weight; BWG, body weight gain; FI, Feed intake.

^b^ D = vitamin D3 (2,760 IU/kg); H25D = vitamin D3 (2,760 IU/kg) + 62.5 mg HyD/ton (full dose); H25D + Ca/P = vitamin D3 (2,760 IU/kg) + 62.5 mg HyD/ton (full dose) + 10%–30% higher Ca&P levels; D + Ca/P = vitamin D3 (2,760 IU/kg) + 10%–30% higher Ca&P levels.

**TABLE 5:** DEXA results for week 17.

Treatment	BMD (g/cm^2^)	BMC (g)	Bone area (cm^2^)	Fat percent (%)	Fat wt (kg)	Muscle percent (%)	Muscle wt (kg)	Total weight (kg)
D	0.219	33.086	151.571	25.278	0.300	74.656	0.823	1.188
H25D	0.211	30.743	145.286	25.483	0.300	74.511	0.877	1.177
H25D + Ca/P	0.217	32.225	148.625	25.175	0.295	74.820	0.878	1.173
D + Ca/P	0.216	31.943	147.286	24.506	0.280	75.494	0.864	1.144
SEM	0.011	1.311	3.584	1.447	0.097	3.523	0.119	0.102
*p*-value	0.956	0.388	0.378	0.883	0.741	0.874	0.801	0.448

^a^ BMD, Bone mineral density; BMC, Bone mineral content.

^b^ D = vitamin D3 (2,760 IU/kg); H25D = vitamin D3 (2,760 IU/kg) + 62.5 mg HyD/ton (full dose); H25D + Ca/P = vitamin D3 (2,760 IU/kg) + 62.5 mg HyD/ton (full dose) + 10%–30% higher Ca&P levels; D + Ca/P = vitamin D3 (2,760 IU/kg) + 10%–30% higher Ca&P levels.

**FIGURE 1:**
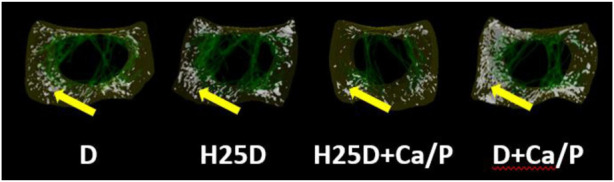
Total porosity within the cortical bone region. 1) Arrow indicating the open pores within the cortical region of the bone. 2) D = vitamin D3 (2,760 IU/kg); H25D = vitamin D3 (2,760 IU/kg) + 62.5 mg HyD/ton (full dose); H25D + Ca/P = vitamin D3 (2,760 IU/kg) + 62.5 mg HyD/ton (full dose) + 10%–30% higher Ca&P levels; D + Ca/P = vitamin D3 (2,760 IU/kg) + 10%–30% higher Ca&P levels. 3) Please refer to Table 3 for definitions of parameters.

However, changes in mineral deposition and 3D structural changes were observed in the femurs. For cortical bone analyses by Micro-CT, the H25D + Ca/P had significantly higher cortical bone volume/tissue volume (BV/TV) compared to the D + Ca/P (Table 6). 25-(OH)D_3_ with higher Ca and P levels (H25D + Ca/P) had significantly lower open pore volume space, open porosity (% cortical tissue volume), total volume of pore space, and total porosity (% cortical tissue volume) in the cortical bone compared to vitamin D_3_ with higher Ca and P levels (D + Ca/P) (*p* < 0.05) (Table 7; Figure 1).

**TABLE 6:** Micro-CT results for bone tissue volume.

Treatment	Total	Trabecular	Cortical
	%	%	%
D	29.963	3.769^b^	95.729^a^
H25D	30.243	3.912^b^	94.794^ab^
H25D + Ca/P	32.032	5.120^a^	95.774^a^
D + Ca/P	31.566	4.435^ab^	93.525^b^
SEM	0.6985	0.5231	0.6324
*p*-value	0.3241	0.0492	0.0384

^a^ D = vitamin D3 (2,760 IU/kg); H25D = vitamin D3 (2,760 IU/kg) + 62.5 mg HyD/ton (full dose); H25D + Ca/P = vitamin D3 (2,760 IU/kg) + 62.5 mg HyD/ton (full dose) + 10%–30% higher Ca&P levels; D + Ca/P = vitamin D3 (2,760 IU/kg) + 10%–30% higher Ca&P levels.

^b^ Please refer to Table 3 for definitions of parameters.

a,b means within a column with different superscripts are significantly different (*p* < 0.05).

**TABLE 7:** Micro-CT results for cortical bone porosity analysis.

Treatment	Volume of closed pores	Closed porosity (percent)	Volume of open pore space	Open porosity (percent)	Total volume of pore space	Total porosity (percent)
Unit	mm^3^	%	mm^3^	%	mm^3^	%
D	0.754	0.887	2.906^b^	3.414^b^	3.660^b^	4.271^b^
H25D	0.760	0.905	3.838^ab^	4.341^ab^	4.598^ab^	5.206^ab^
H25D + Ca/P	0.811	0.933	2.999^b^	3.325^b^	3.811^b^	4.226^b^
D + Ca/P	0.801	0.923	5.168^a^	5.606^a^	5.969^a^	6.475^a^
SEM	0.1285	0.1324	0.4025	0.4012	0.50124	0.4852
*p*-value	0.9956	0.9325	0.0398	0.0358	0.0497	0.0487

^a^ D = vitamin D3 (2,760 IU/kg); H25D = vitamin D3 (2,760 IU/kg) + 62.5 mg HyD/ton (full dose); H25D + Ca/P = vitamin D3 (2,760 IU/kg) + 62.5 mg HyD/ton (full dose) + 10%–30% higher Ca&P levels; D + Ca/P = vitamin D3 (2,760 IU/kg) + 10%–30% higher Ca&P levels.

^b^ Please refer to Table 3 for definitions of parameters.

a,b means within a column with different superscripts are significantly different (*p* < 0.05).

**FIGURE 2:**
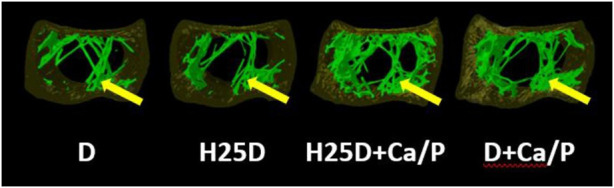
Total pattern factor, matrix, and thickness within the trabecular bone region. 1) Arrow indicating the pattern factor, matrix, and thickness within the trabecular region of the bone. 2) D = vitamin D3 (2,760 IU/kg); H25D = vitamin D3 (2,760 IU/kg) + 62.5 mg HyD/ton (full dose); H25D + Ca/P = vitamin D3 (2,760 IU/kg) + 62.5 mg HyD/ton (full dose) + 10%–30% higher Ca&P levels; D + Ca/P = vitamin D3 (2,760 IU/kg) + 10%–30% higher Ca&P levels. 3) Please refer to Table 3 for definitions of parameters.

The H25D + Ca/P treatment had the lowest trabecular pattern factor (TPF) (*p* = 0.009) and structure model index (SMI) (*p* = 0.031) among the treatments (Table 8; Figure 2). Moreover, H25D + Ca/P had a higher trabecular percentage (trabecular bone/cavity volume%) compared to D and H25D (*p* < 0.05).

**TABLE 8:** Micro-CT results for trabecular bone structural analysis.

Treatment	Trabecular thickness	Trabecular pattern factor	Degree of anisotropy	Connectivity	Connectivity density	Trabecular SMI
	mm	mm^-1^	None	None	mm^-3^	None
D	0.152	5.751^a^	2.189	246.500	1.174	1.632^a^
H25D	0.156	5.834^a^	2.423	271.000	1.298	1.664^a^
H25D + Ca/P	0.159	4.555^b^	2.493	302.571	1.471	1.431^b^
D + Ca/P	0.153	5.323^a^	2.314	390.750	1.830	1.571^ab^
SEM	0.0095	0.2445	0.0986	48.3651	0.2982	0.07426
*p*-value	0.9026	0.02145	0.0721	0.1368	0.2512	0.0452

^a^ D = vitamin D3 (2,760 IU/kg); H25D = vitamin D3 (2,760 IU/kg) + 62.5 mg HyD/ton (full dose); H25D + Ca/P = vitamin D3 (2,760 IU/kg) + 62.5 mg HyD/ton (full dose) + 10%–30% higher Ca&P levels; D + Ca/P = vitamin D3 (2,760 IU/kg) + 10%–30% higher Ca&P levels.

^b^ Please refer to Table 3 for definitions of parameters.

a,b means within a column with different superscripts are significantly different (*p* < 0.05).

The 25-(OH)D_3_ level in the serum was measured in the present study. The treatments with H25D always had the highest 25-(OH)D_3_ level in the serum compared to the other treatments (*p* < 0.0001) (Table 9).

**TABLE 9:** 25-(OH)D_3_ level in the serum.

Treatment	25-(OH)D_3_
	ng/ml
D	28.21^b^
H25D	53.26^a^
H25D + Ca/P	54.31^a^
D + Ca/P	30.18^b^
SEM	2.214
*p*-value	<.0001

^a^ D = vitamin D3 (2,760 IU/kg); H25D = vitamin D3 (2,760 IU/kg) + 62.5 mg HyD/ton (full dose); H25D + Ca/P = vitamin D3 (2,760 IU/kg) + 62.5 mg HyD/ton (full dose) + 10%–30% higher Ca&P levels; D + Ca/P = vitamin D3 (2,760 IU/kg) + 10%–30% higher Ca&P levels.

^b^ MS/LC, mass spectrometry/liquid chromatography.

a,b means within a column with different superscripts are significantly different (*p* < 0.05).

## Discussion

Bone mineral acquisition is organized by a range of factors ranging from genetic determinants and nutritional influences on the hormonal balance, including traditional regulation of mineral homeostasis by vitamin D_3_ (Dusso et al., 2005; Bouillon et al., 2008). Vitamin D_3_ is obtained from dietary sources and is synthesized in the skin by photo-conversion of 7-dehydrocholesterol to vitamin D3, which afterwards undergoes two main modifications (Chen et al., 2020a). Firstly, it is metabolized in the liver to produce the circulating form 25-hydroxyvitamin D_3_ (H25D, calcidiol), which is later converted in the kidney and other tissues including bone by 1α-hydroxylase to generate the active form 1,25-hydroxyvitamin D_3_ (1,25(OH)_2_D_3_, calcitriol) (Chen et al., 2020a). 1,25(OH)_2_D_3_ is the primary hormonal form of vitamin D3 which binds to vitamin D receptor (VDR), inducing a broad range of biological responses (Dusso et al., 2005; Bouillon et al., 2008).

The advantage of H25D during the rearing period is observed more commonly through the bone parameters of pullets such as the cortical and trabecular regions of the bone (Chen et al., 2020a). In the current study, the rearing growth parameters were not affected by H25D. This result agrees with studies reporting that different concentrations and forms of vitamin D_3_ did not affect the growth parameters of pullets (Wen et al., 2019; Chen et al., 2020a; Li et al., 2021). Moreover, the dual-energy X-ray absorptiometry scanning did not detect beneficial changes by vitamin D_3_ and Ca and P in the current study for the rearing period. Similar results were observed in the previous study (Chen et al., 2020a) where at 17 wk, there was no difference in whole-body BMD and BMC while feeding birds with 25D.

With 3D scanning and automatic bone separation process by Micro-CT, the effects of dietary H25D + Ca/P supplementation on pullet bone 3-dimensional structural changes were observed in the present study. In a previous study by Chen et al. (2020a), the H25D increased cortical bone size and porosity significantly but reduced cortical bone density compared to the D treatment alone, suggesting that mineral supplementation or utilization may not be adequate to fill in the larger cortical bone size. For the current study, the primary function of H25D in early bone development was to increase the bone structural size, and to fill the “larger” bone structure with the increased Ca and P in order to close the open pores within the cortical region. This may contribute to bone fracture resistance, as the cortical bone represents 70%–80% of the total bone mass, which is found on the outer surface of bones and its primary function is to support rigidity to the bone, with its relative ratio depending on the function and mechanical load of the respective bone (Digirolamo et al., 2013). It is distinguished by a volumetric fraction (bone mass volume/total volume ratio), which explains its common name of compact bone (Digirolamo et al., 2013). In previous research, the expansion of the cortical bone size with the same amount of BMC resulted in a low-density bone (Chen et al., 2020a). A low BMD is commonly associated with an elevated risk of bone fracture (Ammann and Rizzoli, 2003). A larger cortical region does not necessarily correlate to stronger or more structurally sound bones without having the proper mineral deposition (Estefania et al., 2019).

Inside the cortical cortex, the coupling between osteoblast and osteoclast activities is carried out in the matrix of cortical pores which provide a conduit for the movement of neurovascular structures (Rique et al., 2019). Cortical porosity is considered to be correlated with mechanical properties of cortical bone, manipulating bone strength and risk of fracture (Li et al., 2021). Because larger cavities indicate increased osteoclast recruitment and activity, there is a conjecture that higher bone resorption, either alone or in combination with inhibited osteoblast function, accounts for higher porosity. There is evidence that cortical diameter of pores increases with increased porosity, which can potentially result in decreased overall bone density (Rique et al., 2019). However, decreased porosity and cortical diameter of pores can potentially lead to better overall bone density. In addition, a recent concept of two individual osteoblast types positioned in the cortical bone (Shapiro, 2008), creates another likelihood, that is 1,25(OH)_2_D_3_ selectively suppresses the intracortical osteoblasts coating primary osteons, but without altering surface mesenchymal osteoblasts lining the periosteum. The current study observed a larger cortical bone size with increased pores, which were filled in with elevated levels of calcium (from 30% early on to 10% at later stages) and phosphorus (30% throughout) provided in the diet. This was further observed by the trending volume of closed pores and closed porosity for the H25D + Ca/P treatment, which complements the findings with the lower open pore space. Therefore, the nutritional strategy with H25D + Ca/P during rearing periods have potential to create a more compact and structurally sound bone.

In the present study, higher content of trabecular bone in the H25D treatment was found during the rearing period. The trabecular bone corresponds to 20%–30% of the bone mass, which is in the internal section of the bone (Digirolamo et al., 2013). It has a porous reticular structure with variable and rather low density that provides flexibility and strength to the whole bone structure. It is also distinguished by a relatively low volumetric fraction, offsetting with a surface area nearly twice as large as the compact bone (Digirolamo et al., 2013). Poultry bones were largely based on bone ash, breaking strength, or DEXA (Hester et al., 2004; Castro et al., 2019; Adhikari et al., 2020). These approaches are based on the results of planar morphology or bone mass. Although bone quantity and density are important factors for bone strength (Hester et al., 2004), these parameters do not consider the trabecular architectural changes that are independently related to bone strength (Siffert et al., 1996; Webber et al., 1998). An avian model study determined that over 10% loss of trabecular bone could impact bone strength (Reich and Gefen, 2006), indicating that the integrity of trabecular bone is essential for bone resistance to force. The increase of trabecular bone content within the cavity in the H25D treatment suggested the higher fracture resistance elevated by dietary supplementation. The current findings of a significant decrease in TPF and SMI by the H25D + Ca/P vs. the control D, which was complemented by higher trabecular thickness, number and bone mineral density along with lower separation for the H25D treatment is in agreement with a study (Idelevich et al., 2011). The study observed that inclusion of H25D increased trabecular number and thickness, while having a corresponding decrease in trabecular separation in rats. The study also revealed an increase in overall trabecular bone content with the inclusion of H25D. The correspondence of the SMI, which is an index evaluating whether trabecular bone is rod-like or plate-like, and a smaller value means a more plate-like structure (Hildebrand & Rüegsegger, 1997) and TPF, which is an index evaluating rod-like, plate-like, or honeycomb-like structure, and a small value means a more plate-like to honeycomb-like structure (Hahn et al., 1992), resembles a positive outcome when both parameters are lower as observed in this current study. This correlation of having both SMI and TPF lower also results in increasing overall trabecular structure by increasing connectivity (Chiba et al., 2010; Yokota et al., 2020). Furthermore, for both SMI and TPF, the smaller values indicate the positive plate-like or honeycomb-like structure also results in less osteoporosis cases vs. the rod-like structure increases osteoporosis incidences (Felder et al., 2020).

This study suggests that early supplementation of the combination of H25D with higher level of Ca and P could improve the cortical bone microstructure and have beneficial effects on trabecular bone 3D structural development in pullets. The combination of a higher bio-active forms of vitamin D_3_ and higher levels of Ca and P during rearing periods may become a promising feeding strategy to enhance pullet bone quality.

## Data Availability

The raw data supporting the conclusion of this article will be made available by the authors, without undue reservation.
